# Brief Reviews for Institutional Readers

**Published:** 1913-10-25

**Authors:** 


### BRIEF REVIEWS FOR INSTITUTIONAL READERS.

Certificate Hygiene. Edited by Rev. A. W. Parry, M.A., B.Sc. (London: University Tutorial Press, Limited. 1913. Price 1s. 6d.)

School hygiene in brief summarises the scope of this manual. It is admittedly made up mainly from material taken from Dr. Lyster's "School Hygiene" and Mr. Welpton's "Physical Education," so that it may be imagined there is a sound groundwork therein for the student of hygiene. The book provides all the hygiene required by the Syllabus on the Principles of Teaching for the Acting Teachers' Certificate Examination in 1914 and subsequent years. On such matters as rest and fatigue, accidents, mental deficiencies, ventilation, and so on there is supplied the necessary information to enable the teacher to deal with those points which are continually arising in the course of his daily work. To the intelligent teacher the book should prove of great assistance, and the publishers are to be congratulated on its low price, as well as upon the author's merits.

London Public Health Administration. A Summary. By W. McC. Wanklyn, B.A., M.R.C.S. (London: Longmans, Green and Co. 1913. Price 2s. 6d.)

This little work is a useful summary of public health administration in London. It contains a list of the public health authorities, with an account of their origin, a list of the principal statutes in their chronological order, and a list of the public health services, with the appropriate authorities and the legislation relating thereto. In a very simple and easily referred to arrangement this book presents a conspectus of London public health administration which should prove useful to students reading for the diploma in public health.

